# 905. Risk of Hepatitis B Reactivation in Patients Receiving Ibrutinib: The National VA Cohort

**DOI:** 10.1093/ofid/ofab466.1100

**Published:** 2021-12-04

**Authors:** Ting-Yi Chen, David Jacob, John David Coppin, Chetan Jinadatha

**Affiliations:** 1 Central Texas VA, Austin, Texas; 2 Central Texas Veterans Health Care System, Temple, Texas; 3 Central Texas Veterans Health Care System, Temple, TX, Temple, Texas

## Abstract

**Background:**

Ibrutinib, a bruton tyrosine kinase inhibitor was approved by Food and Drug Administration (FDA) in 2013 and became the first-line treatment for chronic lymphocytic leukemia in 2014. The risk Hepatitis B Virus (HBV) reactivation after initiation of ibrutinib is unclear. Here, we report the results of national Veterans Health Administration (VHA) pharmacy database review estimating the incidence of HBV reactivation after initiation of ibrutinib.

**Methods:**

Veterans who received ibrutinib between Feb 1, 2014 through October 31, 2019 were included in our study. Possible reactivations were identified by change of Hepatitis B Virus surface antigen (HBV sAg), HBV core antibody (Ab) or HBV viral load from no data or negative to positive after starting ibrutinib. Individual chart review was conducted to verify HBV reactivation due to ibrutinib. Cumulative incidence was calculated by identifying HBV reactivation cases among at risk patients, which was defined as prior exposure by positive HBV core Ab regardless of HBV sAg or HBV viral load status. For patients without any HBV serology, an estimated prevalence of HBV exposure in veterans from the literature is used.

**Results:**

A total of 4130 veterans were on ibrutinib during the study period. Of 4130 patients, 1875 patients with HBV core Ab negative and 68 patients on antivirals against HBV prior to ibrutinib were excluded. Among the remaining 2187 patients, there were 170 patients with positive HBV core Ab and 2017 patients without HBV core Ab tested regardless of HBV sAg or HBV DNA status. We used the estimated 13.6% (95%CI 11.5-16.1) of HBV exposure in veterans and estimated that 274 (95%CI 232-325) out of 2017 patients would be at risk of HBV reactivation. Thirty-nine patients were identified to have HBV reactivation after ibrutinib. After detailed review, 7 HBV reactivations were attributable to initiation of ibrutinib. The cumulative incidence of HBV reactivation after ibrutinib was estimated as 1.5% (95% CI 1.4-1.7).

**Conclusion:**

In this large VHA study, we identified 7 cases of HBV reactivations among 444 at risk patients. The cumulative incidence of HBV reactivation after ibrutinib was 1.5% in patients with prior HBV exposure with positive HBV core Ab irrespective of HBV sAg or HBV DNA status, indicating a moderate risk of HBV reactivation.

**Disclosures:**

**All Authors**: No reported disclosures

